# Preface to special issue on plenary presentations of Second International Conference on Rail Transportation

**DOI:** 10.1007/s40534-022-00289-8

**Published:** 2022-09-07

**Authors:** Wanming Zhai

**Affiliations:** Southwest Jiaotong University, Chengdu, 610031 China

The Second International Conference on Rail Transportation (ICRT), initiated and hosted by Southwest Jiaotong University, completed its agenda after two days of academic reports and seminars, and came to a successful end on 6 July 2021, in Chengdu, China. The conference chairman was Professor Wanming Zhai from Southwest Jiaotong University. The conference received tremendous support from multiple co-sponsors including National Natural Science Foundation of China, Transportation & Development Institute of ASCE, National Innovation Center of High Speed Train of China, University of Illinois at Urbana-Champaign, Tongji University, Delft University of Technology, China Academy of Railway Sciences, Royal Institute of Technology (KTH), Central South University, Politecnico di Milano, Central Queensland University, Beijing Jiaotong University and University of Technology Sydney.

The 2nd ICRT covered a variety of railway research fields such as rail vehicles, infrastructures, traction power, operation and maintenance, environment impacts, sustainable development, etc. Affected by the COVID-19 epidemic, the conference was held in a combination of online and live ways. As one of the largest gatherings of railway researchers, engineers, and technicians, this conference has attracted more than 70 well-known experts to join the conference committees, over 300 Chinese representatives to meet in Chengdu, and thousands of scholars from 22 countries to participate online. After a rigorous peer review process, the conference has accepted 120 full-length papers from 18 countries, and 96 oral presentations have been selected in the conference program.

Particularly, nine distinguished railway experts from different research fields were invited to deliver plenary presentations, who shared the state-of-the-art academic research and engineering practices. This Special Issue of *Railway Engineering Science* is published for the extended versions of six plenary presentations, which are related to design of locomotive, wheel-rail interaction, train-bridge interaction, railway track structure and subgrade. The following are brief introductions of the six articles:

1. Prof. Maksym Spiryagin from Central Queensland University, Australia, systematically presented the problems, assumptions and solutions in locomotive design, traction and operational studies and summarized the existing major challenges in the future developments of locomotive traction science and digital twins.
2. The research team led by Professors Yunmin Chen and Xuecheng Bian of Zhejiang University, China, focused on the mud pumping problem in high-speed railway. On the basis of the numerical simulation, in-situ soil core test, and full-scale physical model of ballastless track, the formation of mud pumping in roadbed of ballastless track was revealed, and the effectiveness and engineering application of the polyurethane injection on mud pumping remediation was verified.
3. Prof. Buddhima Indraratna from University of Technology Sydney, Australia, presented the mechanical behaviors of four recycled rubber products applied in railways and developed a rheological track model to reveal the energy dissipation capacity of the track with these applications, which may promote environmental sustainability and more affordable ballasted tracks.
4. Prof. António Gomes Correia from University of Minho, Portugal, proposed a geomechanics classification for subgrade coupling the stiffness (resilient modulus) and permanent deformation behaviour, which could be a potentially useful tool in the evaluation and modelling of the foundation of railway structures.
5. The research team led by Prof. Zili Li of Delft University of Technology, the Netherlands, presented new insights into the friction modifiers (FMs) by experimentally investigating the effects of two types of top-of-rail FMs, and their application dosages on wheel–rail dynamic interactions with various angles of attack.
6. Prof. Yongle Li from Southwest Jiaotong University, China, presented a comprehensive literature review of research works on train–bridge systems under external excitations including crosswinds, waves, collision loads, and seismic loads, and provided some valuable suggestions for further research on train–bridge coupling vibrations under external excitations.

These papers collectively present the recent progress, new insights and in-depth discussions in the aspect of essential challenging issues from rail transportation industry, which are expected to provide useful guidance and inspiration for the above research subjects.

We would like to sincerely appreciate all contributing authors, involved peer reviewers, management and technical teams for their effort, time and collaboration to guarantee the high-quality publications of this Special Issue.

